# Influence of age on muscle sympathetic response to dynamic exercise

**DOI:** 10.1113/EP091009

**Published:** 2023-01-12

**Authors:** Catherine F. Notarius, John S. Floras

**Affiliations:** ^1^ Clinical Cardiovascular Physiology Research Laboratory University Health Network and Sinai Health Toronto Ontario Canada; ^2^ Faculty of Kinesiology and Physical Education University of Toronto Toronto Ontario Canada; ^3^ Department of Medicine University of Toronto Toronto Ontario Canada

We read with interest the paper from the Katayama laboratory, entitled ‘Sympathetic vasomotor outflow during low‐intensity leg cycling in healthy older males’ (Katayama et al., 2022). To investigate the mechanism responsible for the exaggerated inhibition of contralateral fibular muscle sympathetic nerve activity (MSNA) during mild and moderate one‐leg cycling that we had detected in older, compared with young healthy subjects (Notarius et al., 2019), these authors added an estimate of central venous pressure and recorded MSNA from the radial nerve, during two‐leg cycling, in young and elderly men. The increases in estimated central venous pressure elicited by low‐intensity cycling in the two cohorts were similar in their experiments. Also, they observed, as did we, similar inhibition of MSNA burst frequency during mild‐ and moderate‐intensity exercise in the two cohorts. Their interpretation of these central venous pressure and burst frequency data was that in healthy older males, muscle pump‐induced loading of the cardiopulmonary baroreflex during cycling was preserved.

Importantly, our findings differ with respect to the influence of such exercise on heart rate, hence MSNA burst incidence. In contrast to Katayama et al. (2022), heart rates of our older cohort rose less (*P* = 0.03), and their MSNA fell further (−19 ± 2 vs. −11 ± 2 bursts/100 heart beats; *P* = 0.01), indicating augmented reflex or centrally mediated sympathetic inhibition.

Several factors might account for these discordant findings. Katayama et al. (2022) studied men exclusively and, considering the variability of resting MSNA across the adult lifespan in both males and females (Keir et al., 2020) and that MSNA was measured in only nine in each age group (we enrolled 18 in each, with a 3:1 male‐to‐female ratio), their protocol might have been underpowered to detect true differences. Exercise capacity was not measured, but both groups were deemed untrained, whereas our young and older participants were matched for resting blood pressure, heart rate and fitness level, measured directly and expressed as a percentage of predicted peak oxygen uptake (mean in older group, 110%, and in the young, 114% of that predicted for their age). The mean exercise work rate also differed between their groups (10 W in old, 6 W in young). As these authors are aware, inhibition of MSNA during low‐intensity cycling becomes less with increasing absolute exercise intensity (Saito & Mano, 1991). Thus, the lower workload in the young might have obscured the detection of age differences. Recording from the radial nerve is unlikely to account for these differences; a recent report with Dr Katayama as co‐author demonstrated good coherence of burst frequency and incidence between limbs during various modes of exercise (Lee et al., 2022).

Katayama et al. (2022) cite prior work concluding that the arterial baroreflex control of sympathetic vasomotor discharge is preserved in older individuals, but did not develop this concept further in the analysis of their exercise data. We attributed the greater reduction in MSNA burst incidence in our older group (mean age, 57 ± 2 years), in part, as reflexively appropriate to their augmented pressor response to exercise from a similar baseline (+21 ± 5 vs. +10 ± 1 mmHg, systolic). In the experiments by Katayama et al. (2022), systolic pressure, at rest, was 14 mmHg higher in the older group and increased by 24 mmHg in the older men (mean age 72 ± 3 years) and by 11 mmHg in the young men.

By measuring estimated central venous pressure, the authors settle nicely the question of whether the central translocation of blood via the muscle pump (stimulating the sympatho‐inhibitory cardiopulmonary baroreflex) changes with age. Here, they found no difference between young and old. However, the most plausible interpretation for their neutral findings with respect to MSNA burst incidence, namely attenuated arterial baroreflex regulation within their (on average, 15 years) older cohort of men, secondary to diminished conduit artery compliance, appears to have been overlooked.

## CONFLICT OF INTEREST

None declared.

## FUNDING

Canadian Institutes for Health Research: John S. Floras, PJT148836; Heart and Stroke Foundation of Canada (HSF): John S. Floras, T4938, NA6298 Dr. Floras has received grants from the Canadian Institutes for Health Research (CIHR) (PJT148836) and the Heart and Stroke Foundation of Canada (T4936, NA6298).
